# On the value of debate in medical school

**Published:** 2017-02-24

**Authors:** Ariane Litalien

**Affiliations:** McGill University, Quebec, Canada

Recently, I took to Facebook to ask my classmates a tough question: “is it acceptable to pay someone to receive a slight academic advantage in medical school?” For example, should we be allowed to offer money for detailed notes in preparation for an exam? Is it acceptable to pay money for a better spot in line for an anatomy examination, one that would allow us to take the test earlier and leave us more time to study for the next midterm? Is it inclusive to offer money for things that may slightly improve your grades, given that some students with lower incomes may be unfairly penalized in these situations? Or should the market forces of supply and demand prevail, as they do in most other aspects of life?

To my surprise, I was immediately rebuffed—and for reasons I had not anticipated. Instead of answering the question I asked and weighing in on the arguments presented, instead of completely demolishing my opinion (even that would have been better), people asked: “Why does every little thing have to be the source of debate?”

I strongly believe that the medical student has an obligation to consider—and one could even argue, to intervene in—situations where those who are economically disadvantaged may be directly or indirectly discriminated against in other ways, such as admission to medical school. To refuse to do so is not only contrary to the CanMEDS role of Health Advocate, but it also profoundly fails the altruistic foundations of medicine. And I was mostly hoping to engage in a debate.

More broadly, as an intellectual, the medical student should seek debates as a way to further his or her knowledge and to improve not only his or her personal actions, but also the larger context within which these arguments take place. No real positive change has ever taken place in the absence of debate or controversy, whether it was the end of slavery in the United States or the acceptance that the earth is round. Debates allow us to move closer to the truth, help us become better people, teach us how to keep an open-mind, and encourage us to put ourselves in other people’s shoes—skills that every good clinician should exhibit.

Finally, as a citizen of the world, the medical student has an obligation towards peers and society in general to consider whether his or her actions are harmful, to create inclusive environments, and to contribute to a leveled-playing field, both within and outside the classroom.

And so yes, debates about “every little thing” may seem excessive, but debates about the big things—what kind of values we should embrace as medical students, how we can make our classroom environment more inclusive, and whether some of our practices contribute to social inequality (and poor health of our patients)—those are the big things we should absolutely debate. It can certainly be unsettling to realize that we, even unintentionally, contribute to oppression in one way or another, yet our knee-jerk reaction should be to engage in debate about our behaviors and, if appropriate, to adjust our practices; instead it seems we are just fleeing from debate altogether.

